# Breaking the Perfluorooctane Sulfonate Chain: Piezocatalytic Decomposition of PFOS Using BaTiO_3_ Nanoparticles

**DOI:** 10.1002/smsc.202400337

**Published:** 2024-08-28

**Authors:** Andrea Veciana, Sarah Steiner, Qiao Tang, Vitaly Pustovalov, Joaquin Llacer‐Wintle, Jiang Wu, Xiang‐Zhong Chen, Trust Manyiwa, Venecio U. Ultra, Beltzane Garcia‐Cirera, Josep Puigmartí‐Luis, Carlos Franco, David J. Janssen, Laura Nyström, Samy Boulos, Salvador Pané

**Affiliations:** ^1^ Institute of Robotics and Intelligent Systems ETH Zurich Tannenstrasse 3 CH 8092 Zurich Switzerland; ^2^ Institute of Optoelectronics State Key Laboratory of Photovoltaic Science and Technology Shanghai Frontiers Science Research Base of Intelligent Optoelectronics and Perception International Institute of Intelligent Nanorobots and Nanosystems Fudan University Shanghai 200433 P. R. China; ^3^ Yiwu Research Institute of Fudan University Yiwu 322000 Zhejiang P. R. China; ^4^ Department of Earth & Environmental Science Faculty of Science Botswana International University of Science and Technology Plot 10071 Palapye Botswana; ^5^ Departament de Ciència de Materials i Química Física Institut de Química Teòrica i Computacional Universitat de Barcelona 08028 Barcelona Spain; ^6^ Institució Catalana de Recerca i Estudis Avançats (ICREA) Pg. Lluís Companys 23 08010 Barcelona Spain; ^7^ Department Surface Waters Eawag: Swiss Federal Institute of Aquatic Science & Technology 6047 Kastanienbaum Switzerland; ^8^ Department of Health Sciences and Technology Laboratory of Food Biochemistry ETH Zurich Schmelzbergstrasse 9 8092 Zürich Switzerland

**Keywords:** barium titanate, defluorination, degradation, environmental remediation, per‐ and polyfluoroalkyl substances, perfluorooctane sulfonate, piezocatalysis

## Abstract

Per‐ and polyfluoroalkyl substances (PFAS) pose significant environmental and health risks due to their ubiquitous presence and persistence in water systems. Herein, the efficacy of piezocatalysis using barium titanate nanoparticles under ultrasound irradiation for the degradation and defluorination of perfluorooctane sulfonate (PFOS) in water is investigated. The research demonstrates a substantial 90.5% degradation and 29% defluorination of PFOS after 6 h of treatment, highlighting the potential of piezocatalysis as a promising approach for PFAS degradation. Additionally, the quantification of degradation products elucidates the transformation pathways of PFOS, suggesting a stepwise chain‐shortening mechanism. The findings underscore the importance of continued research in optimizing piezocatalytic processes and exploring synergistic approaches with other advanced oxidation methods to effectively address PFAS contamination challenges. These efforts are essential for advancing sustainable water treatment strategies and mitigating the environmental and health hazards associated with PFAS contamination.

## Graphical abstract

This study investigates, for the first time, the piezocatalytic degradation of perfluorooctane sulfonate (PFOS) in water using BaTiO_3_ nanoparticles activated by ultrasound. Results show a significant 90.5% degradation and 29% defluorination of PFOS after 6 h, highlighting piezocatalysis as a promising approach for addressing the contamination of forever chemicals in aquatic environments.

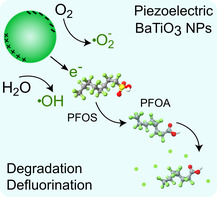

## 1 Introduction

**Figure 1:**
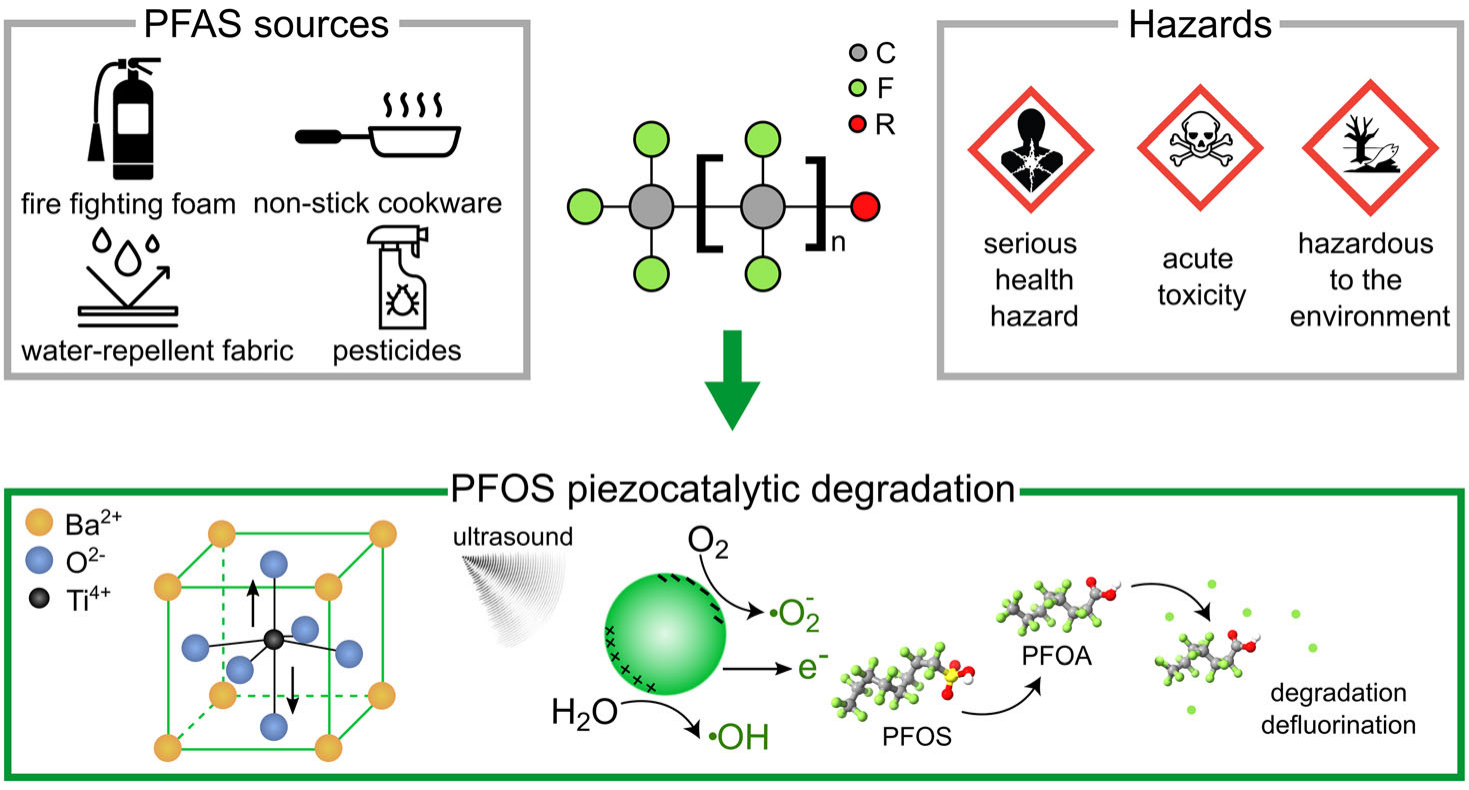
Illustration depicting common sources of PFAS alongside corresponding hazards, and a schematic diagram illustrating the piezocatalytic degradation of PFOS.

Per‐ and polyfluoroalkyl substances (PFAS) represent a group of synthetic chemicals employed in various industrial and consumer products for decades. Known for their thermal and chemical stability, PFAS are denoted as “forever chemicals” for their enduring presence in the environment.^[^ ^1^ ^]^ PFAS are characterized by a hydrophobic fluorinated carbon chain linked to a hydrophilic functional group, as depicted in **Figure** **1**. Their highly stable C—F bond provides PFAS a superior chemical stability, making them instrumental in several applications, including nonstick cookware, water repellent fabrics, and firefighting foams.^[^ ^2^, ^3^ ^]^ However, growing environmental concerns have prompted stringent regulations, including the restriction of production and use of PFAS such as perfluorooctanoic acid (PFOA) and perfluorooctane sulfonate (PFOS) under the 2009 Stockholm Convention.^[^ ^4^ ^]^ In 2019, the convention imposed a general ban on the production of PFOA and its precursors.

Despite regulatory efforts, the widespread use of PFAS has resulted in their release into the environment, where they can persist for many years and spread over long distances.^[^ ^3^, ^5^ ^]^ PFAS have been detected in soil, water, wildlife, and human blood and tissues.^[^ ^6^, ^7^ ^]^ In Switzerland, where 80% of drinking water originates from groundwater, National Groundwater Monitoring NAQUA reveals that the sum of 26 PFAS species present in ground water exceed the established limits proposed by the European Commission of 0.0044 μg L^−1^ on 25% of the monitoring sites.^[^ ^8^ ^]^ Moreover, the potential health risks associated with PFAS contamination, such as increased cancer risks, thyroid dysfunction, and reproductive disorders, further emphasize the urgency of establishing robust and comprehensive water treatment strategies to safeguard both environmental ecosystems and human health.^[^ ^9^, ^10^, ^11^ ^]^

As a result, there has been a growing effort to regulate PFAS and reduce their release into the environment. This has included calls for increased monitoring and reporting of PFAS, as well as efforts to develop technologies for the removal of PFAS from wastewater. It is estimated that the annual cost to eliminate PFAS from the environment at the same rate as its current production and usage is equivalent to the entire current global GDP of 106 trillion USD.^[^ ^12^ ^]^ This has prompted investigation of alternative technologies for the treatment of PFAS‐contaminated water. Physical separation processes, such as carbon adsorption, membrane filtration, and ion exchange, can remove PFAS from water; however, they transfer and concentrate the PFAS from one phase to another instead of degrading them.^[^ ^13^, ^14^ ^]^ In contrast, several advanced oxidation methods such as photocatalysis, sonochemical oxidation, and ozonation have reported the successful defluorination and degradation of various PFAS using highly reactive oxygen species (ROS).^[^ ^15^ ^]^

Amongst these, piezocatalysis, a well‐established field, has been extensively investigated for the degradation of organic pollutants in water. In this approach, piezoelectric materials experience an external strain, typically induced by mechanical forces such as ultrasound irradiation to generate ROS. Several piezoelectric materials such as BaTiO_3_, BiFeO_3_, ZnO, and Bi_4_Ti_3_O_12_ with distinct size and morphology have demonstrated their effectiveness in degrading a wide range of organic pollutants.^[^ ^16^, ^17^, ^18^, ^19^, ^20^, ^21^, ^22^, ^23^ ^]^ Additionally, piezocatalysis can be combined with photocatalysis to overcome the challenges of low light penetration and charge recombination. Furthermore, in the realm of renewable energy sources, mechanical energy emerges as a versatile option, as it can be harnessed from various sources such as water flow, bubbling, and vibrations.

Recent research has shown that piezocatalysis for PFOA degradation could be conducted in the solid phase.^[^ ^24^, ^25^ ^]^ Our contribution aims to broaden this research by specifically focusing on PFOS decomposition in the liquid phase. Our objective is to evaluate the potential degradation and defluorination of PFOS in water using BaTiO_3_ piezocatalytic nanoparticles under ultrasound irradiation (Figure 1).

## 2 Results and Discussion

### 2.1 Material Characterization

**Figure 2:**
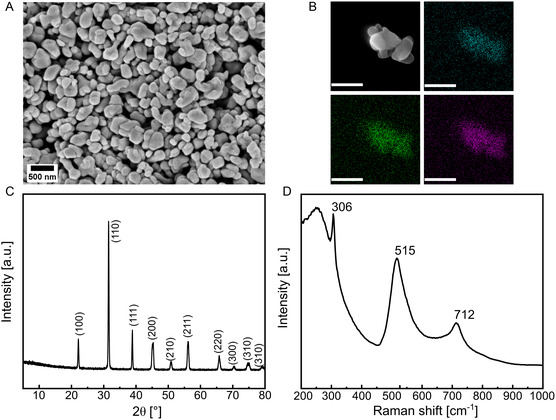
A) An SEM image of BTO nanoparticles. B) EDX analysis with elemental mapping revealing barium (green), titanium (purple), and oxygen (blue) (scale = 500 nm). C) XRD pattern of BTO nanoparticles showcasing the crystal structure. D) Raman spectra providing insights into the formation of the piezoelectric tetragonal phase.

Barium titanate (BTO) nanoparticles were synthesized using a two‐step hydrothermal method.^[^ ^17^ ^]^ After the first step, a distinctive nanowire morphology of the H_2_Ti_3_O_7_ precursor is observed, as shown in the scanning electron microscopy (SEM) image in Figure S4 (Supporting Information). Subsequently, after the second hydrothermal step, the formation of BTO nanoparticles with an approximate size of 200 nm was achieved (**Figure** **2**A). As shown in Figure 2B, energy‐dispersive X‐ray spectroscopy (EDX) mapping was employed to validate the particles composition, confirming the presence of Ba, Ti, and O. Furthermore, to confirm the piezoelectric properties, the nanoparticles’ crystal structure was verified by X‐Ray diffraction (XRD), revealing a pure perovskite structure with characteristic diffraction peaks in agreement with JCPDS No. 81‐2203 (Figure 2C). The peak splitting of the (002) and (200) planes at 2*θ* ≈ 45° further confirms that the nanoparticles display a tetragonal phase, which confirms the ferroelectric nature of BTO (see Figure S5, Supporting Information). The formation of the tetragonal phase was further supported by Raman spectroscopy, presented in Figure 2D, depicting sharp peaks at 305, 515, and 715 cm^−1^.^[^ ^26^ ^]^ Additional insights into the purity of BTO nanoparticles were obtained through X‐ray photoelectron spectroscopy (XPS) analysis. The full XPS spectrum revealed the presence of Ba, Ti, and O elements, as illustrated in Figure S6 (Supporting Information). High‐resolution spectra of Ba 3d revealed two split peaks: Ba 3d5/2 at 778 eV and Ba 3d3/2 at 793.4 eV, corresponding to Ba^2+^ (refer to Figure S6b, Supporting Information).^[^ ^27^, ^28^ ^]^ Similarly, the Ti 2p spectra exhibited two distinct peaks at 457.5 and 463.3 eV, representing Ti 2p_3/2_ and Ti 2p_1/2_ of Ti^4+^ (see Figure S6c, Supporting Information).^[^ ^29^ ^]^ Furthermore, the O 1s spectrum displayed a broad peak at 528.8 eV, attributed to lattice oxygen and surface‐absorbed oxygen (Figure S6d, Supporting Information).^[^ ^27^ ^]^

### 2.2 Piezocatalytic Degradation of PFOS

The BTO nanoparticles, tailored for their piezoelectric properties, were subject to ultrasonic irradiation (Sonocool 255) operating at a frequency of 35 kHz and maximum power of 135 W while maintaining a constant temperature of 24 °C to induce deformation and external strain. This activation step leverages the piezoelectric characteristics of BTO nanoparticles. By doing so, it facilitates their role as catalysts, generating an electrical potential on the surface of the nanoparticles that serves as a trigger to generate ROS. Consequently, this process leads to the degradation of organic micropollutants, such as PFOS, present in water. For the sake of clarity, note that one must differentiate between mineralization, degradation, and defluorination of PFAS in the context of our study. Degradation involves the transformation of PFAS molecules into other compounds, regardless of defluorination.^[^ ^30^ ^]^ Mineralization refers to the complete defluorination, irrespective of whether the carbon is fully oxidized.^[^ ^31^ ^]^ While defluorination specifically refers to the release of inorganic fluorine atoms from PFAS molecules, mineralization does not necessarily occur.

**Figure 3:**
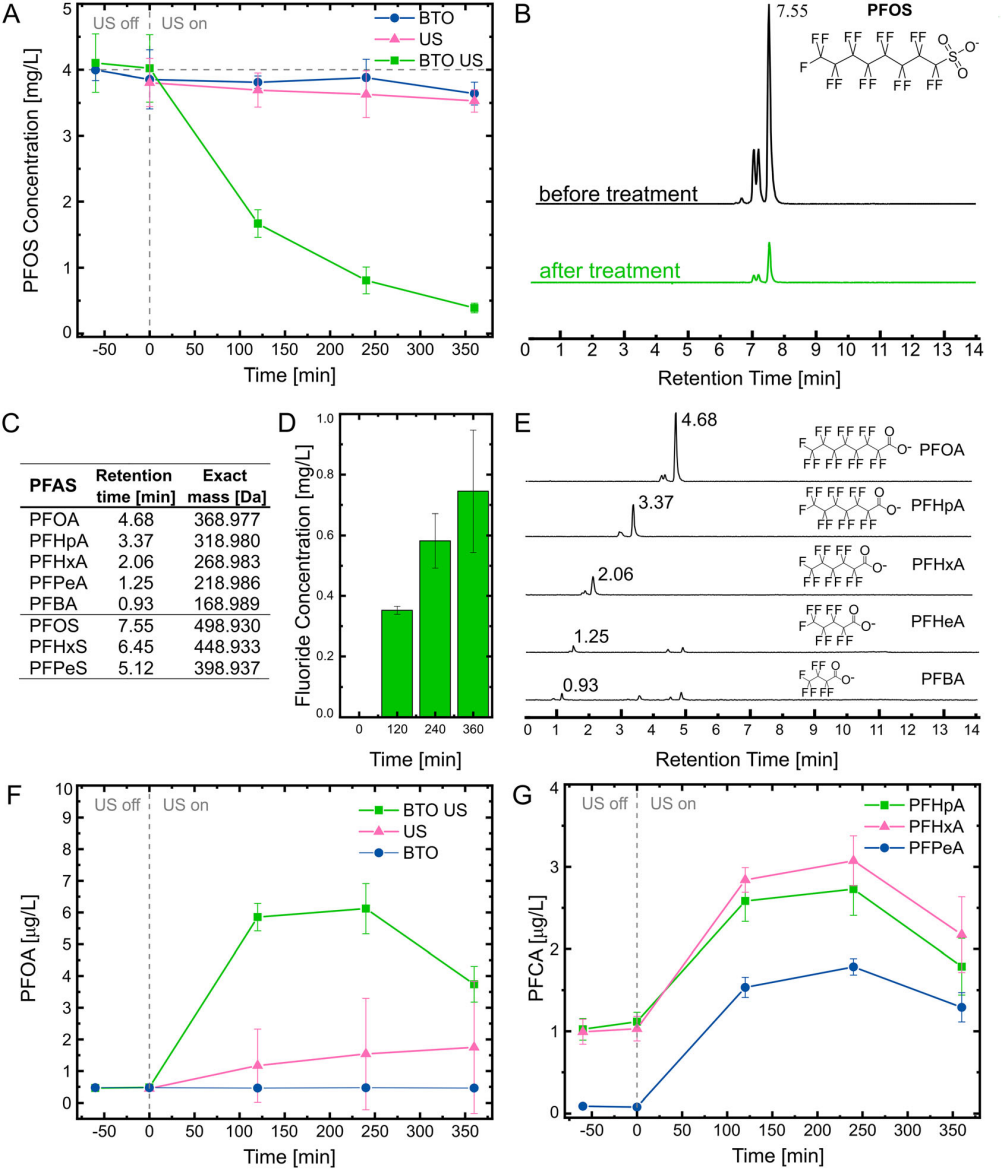
A) Time course of PFOS piezocatalytic degradation in the presence of BTO nanoparticles and controls. B) Extracted ion chromatogram of PFOS before and after treatment. C) Retention time and exact mass of deprotonated PFOS and PFCAs (the latter detected as decarboxylated ions). D) Fluoride concentration over treatment time. E) Extracted ion chromatograms of the detected degradation products after 6 h of treatment representing the depicted corresponding carboxylate species. F) Time course of PFOA production (and later decomposition) under US in the presence of BTO nanoparticles, and G) time course of shorter chain intermediates PFHpA (C7), PFHxA (C6), and PFPeA (C5). The error bars indicate mean ± SD, *n* = 3.

To ensure the efficiency of the piezocatalytic process, initial optimization studies were conducted to determine the optimal catalyst dosage and ultrasound power. As depicted in Figure S7 (Supporting Information), this optimization revealed that the optimal catalyst dosage was found to be 1 mg mL^−1^. In agreement with previous research, the results indicate that increasing the concentration of the catalyst beyond 1 mg mL^−1^ does not necessarily contribute to a further enhancement of the reaction.^[^ ^32^ ^]^ Moreover, the ultrasound power stands out as a critical parameter in piezocatalysis, playing a pivotal role in enhancing the decomposition efficiency. However, a tradeoff exists between achieving faster degradation and ensuring that no unintended sonocatalysis is involved in the process.^[^ ^33^ ^]^ This balance is particularly evident in Figure S8 (Supporting Information), where it is observed that employing 100% of the total power resulted in a gradual decrease of PFOS concentration over time for the control group without the catalyst. Conversely, at 75% power, as illustrated in **Figure** **3**A (dashed lines), no significant degradation occurred in the absence of the catalyst. To provide a clear understanding of the individual effects of piezocatalysis, a 75% ultrasound power was selected for the PFOS degradation experiments.

Remarkably, as depicted in Figure 3A, following 6 h of ultrasound treatment, the PFOS concentration exhibited a substantial reduction from the initial 4 mg L^−1^ to 0.38 mg L^−1^, attaining 90.5% removal. As shown in Figure 3B, the extracted ion chromatogram of PFOS at a retention time of 7.55 min clearly illustrates a notable difference in peak intensity before and after treatment. In addition, the corresponding degradation kinetics fitted well with the pseudo‐first‐order model (described in Supporting Information), revealing a degradation rate of *k* = 0.06 min^−1^ (Figure S9, Supporting Information). Note that the initial concentration is notably high, considering that PFOS is typically detected in water at concentrations ranging from nanograms per liter to micrograms per liter.^[^ ^34^ ^]^ However, an initial large concentration was deliberately selected to facilitate analytical methods for detecting degradation products listed in Figure 3C. Nevertheless, as demonstrated in Figure S10 (Supporting Information), this technique is also effective in decomposing PFOS at an order of magnitude lower concentration (0.4 mg L^−1^), by achieving a 72% PFOS removal.

### 2.3 Quantification of Degradation Products

To further corroborate the defluorination of PFOS via piezocatalysis, the fluoride (F^−^) concentration was monitored over time via ion chromatography, as depicted in Figure 3D. Considering that 17 moles of F^−^ should theoretically be released for each mole of PFOS, the defluorination of ≈90% of PFOS after 6 h of treatment should generate 2.32 mg L^−1^ of F^−^. However, 0.74 mg L^−1^ of F^−^ ions was produced, resulting in an overall defluorination rate of 29%, calculated using Equation S1 (Supporting Information). Regarding the decomposition of PFOS, note that the defluorination ratio is considerably lower compared to the PFOS degradation ratio. This suggests that the transformation of PFOS does not result in complete mineralization. Therefore, preventing secondary pollution arising from the formation of shorter intermediate chains during treatment remains critical. Consequently, the quantification of the degradation products not only ensures a comprehensive understanding of the degradation mechanism but also serves as a crucial step in preventing unintended environmental consequences. PFOS and PFOA stand out as the most frequently detected PFAS in diverse environmental settings, with PFOS consisting of an eight‐carbon chain and a sulfonic acid functional group.^[^ ^35^ ^]^ Figure 3F illustrates the concentration increase of PFOA during treatment, suggesting that the initial decomposition step involves the cleavage of the sulfonate end group. This observation aligns with the lower bond energy of the sulfonate end of the PFOS molecule compared to the C—F or C—C bonds, making direct defluorination via nucleophilic substitution much more challenging. Moreover, considering that the C—S bond has a lower bond energy than the C—C bond, and there is a greater distance between the C and S atoms, C—S scission becomes a more favorable pathway.^[^ ^36^ ^]^ Importantly, the absence of shorter chain perfluoroalkyl sulfonic acids during the screening of degradation products corroborates the desulfonation process.

After 4 h of treatment, a subsequent decrease in PFOA concentration is observed indicating a stepwise chain‐shortening degradation pathway. As presented in Figure 3E, the extracted ion chromatograms after 6 h of treatment depict a clear formation of shorter chain perfluorinated carboxylic acids (PFCAs), with primary intermediates identified as perfluoroheptanoic acid (PFHpA, C7), perfluorohexanoic acid (PFHxA, C6), perfluoropentanoic acid (PFPeA, C5), and perfluorobutanoic acid (PFBA, C4). Notably, no fluorotelomer carboxylic acids were detected during mass spectrometry (MS) screening, ruling out the possibility of a degradation pathway involving H/F exchange. As shown in Figure 3G, while the observed trend in the concentration of intermediate products shows a reduction after four hours of treatment, it is essential to highlight that the concentration of these products is three orders of magnitude lower than that of the parent PFOS. The absence of shorter chain PFCAs in the initial PFOS solution suggests that all detected compounds result from the piezocatalytic degradation. Nonetheless, it is important to note that the total mass balance of the reaction remains incomplete.

The fluorine mass balance is composed of organic fluorine from the detected degradation products and remaining parent compound, free F^−^ ions, and unidentified fluorine compounds. Free inorganic fluoride was measured directly, whereas the organic fluorine found in the liquid phase was estimated using the final concentrations of PFOS and the identified products. Of the total fluorine that was initially present, the remaining PFOS and degradation products accounted for 10% at the end of the treatment, plus the 29% free F^−^. The unaccounted 61% may originate from volatile organofluorine compounds mostly in the gas phase, and possible F^−^ sorbed on the surface of the BTO nanoparticles, as further discussed in Section 2.5.

### 2.4 PFOS Removal Mechanism

Piezoelectric materials, such as BTO, exhibit a non‐centrosymmetrical crystal structure. As a consequence, under mechanical deformation, the piezomaterial becomes electrically polarized. The polarization induced by mechanical stress triggers the separation of holes (h^+^) and electrons (e^−^). Hence, a potential difference exists across the material (Equation (1)). The magnitude of this piezoelectric potential is crucial, as it must meet or exceed the redox potential required for the desired chemical reaction to occur.^[^ ^37^ ^]^ As illustrated in Equation (2) and (3), the free electrons oxidize water to produce hydroxyl radicals (•OH), while holes reduce dissolved O_2_ to generate superoxide radicals (O_2_^•−^). While both radicals are considered predominant active species for the degradation of organic pollutants, O_2_^•−^ may indirectly contribute to the degradation by serving as a crucial source for •OH formation rather than directly participating in the degradation process. The formation of •OH is found to follow two pathways: the reaction of holes with water (Equation (2)) and the two‐electron reduction of •O_2_^−^ as described in Equation (4) and (5).

(1) $${\text{BaTiO}}_{3}+\text{ US }\to {\text{ BaTiO}}_{3}\;(e^{-}+{\text{ h}}^{+})$$

(2) $$h^{+}+{\text{ H}}_{2}\text{O }\to •\text{OH }+{\text{ H}}^{+}$$

(3)

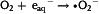



(4)

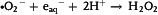

(5) $$H_{2}O_{2}\to •\text{OH }+•\text{OH}$$

**Figure 4:**
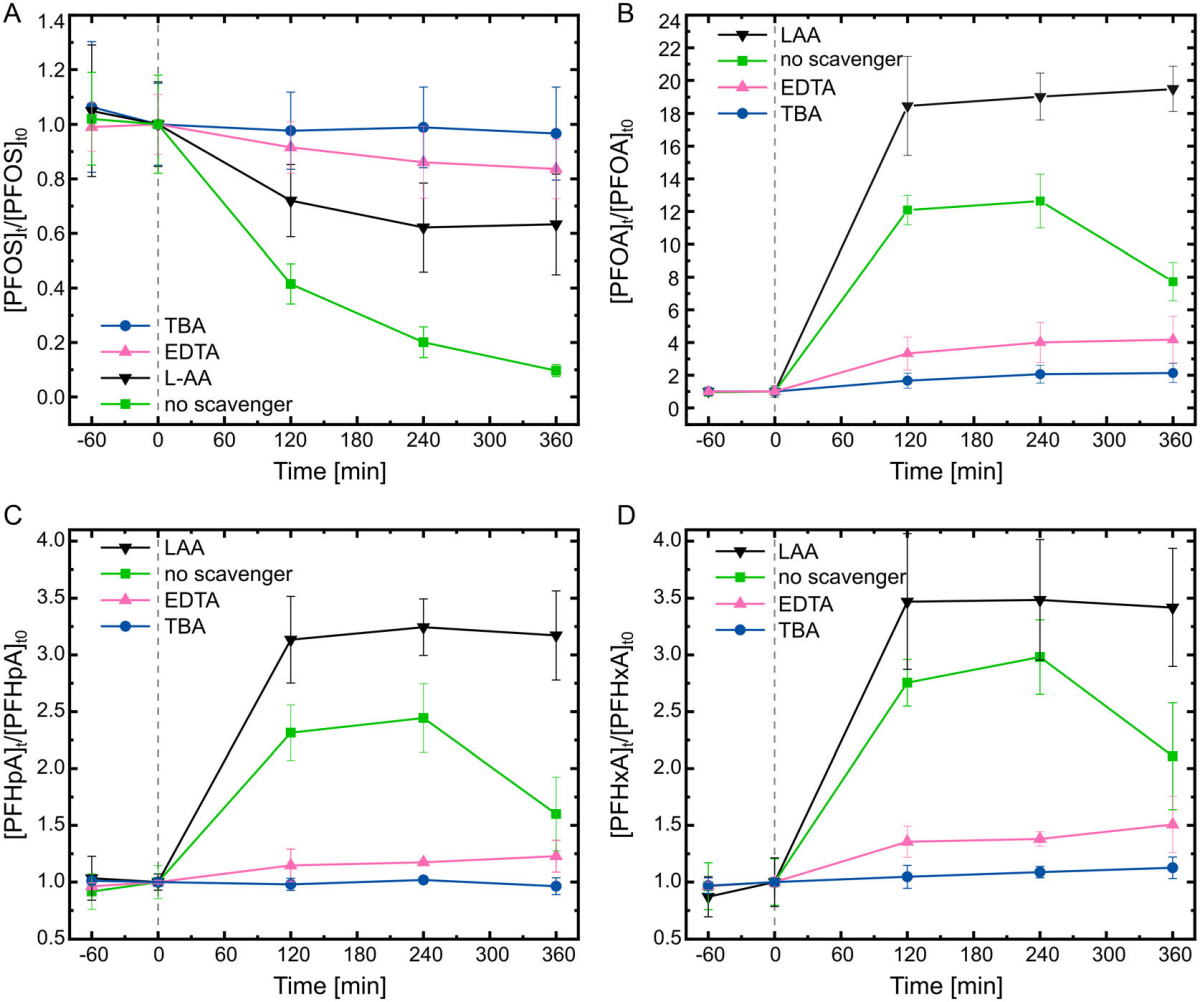
A) Piezocatalytic decomposition of PFOS in the presence of scavenging agents TBA, EDTA, and LAA, trapping •OH, h^+^, and •O_2_^−^, respectively. Time course of detected shorter chain intermediates: B) PFOA (C8), C) PFHpA (C7), and D) PFHxA (C6). The error bars indicate mean ± SD, *n* = 3.

Previous studies have shown that BTO piezocatalysts can generate both reactive species, where the concentration of generated •OH is observed to be significantly higher than that of •O_2_^−^.^[^ ^16^ ^]^ To examine the main reactive species involved in the PFOS degradation, the piezocatalytic reaction was assessed in the presence of scavenging agents. *Tert*‐butyl alcohol (TBA), ethylenedinitrilotetraacetic acid disodium salt dihydrate (EDTA), and l‐ascorbic acid (LAA) were used as common scavengers to quench •OH, H^+^, and •O_2_^−^, respectively.^[^ ^38^, ^39^ ^]^ As shown in **Figure** **4**A, the results show a significant decrease in degradation efficiency after adding TBA, emphasizing the crucial role of •OH. The inhibiting effect was less pronounced with LAA, suggesting an auxiliary role of •O_2_^−^. Moreover, the degradation efficiency showed a decrease, albeit not complete suppression, after adding EDTA, indicating that the formation of •OH partly originates from H^+^ and •O_2_^−^.

Given that the PFOS molecule is inert to hydroxyl radical oxidation, it is plausible that the initial degradation step involves an attack by hydrated electrons on the C—S bond, cleaving the sulfonate terminal group and forming a •C_8_F_17_ radical.^[^ ^40^, ^41^, ^42^ ^]^ Subsequently, as depicted in Figure S11 (Supporting Information), the recombination with an •OH forms an intermediate perfluorooctanol. The final step in the transformation of PFOS into PFOA also involves a hydroxyl radical, an aqueous electron, and a water molecule, which convert perfluorooctanoyl fluoride into PFOA. This process underscores the crucial role of electron transfer in the initial stages of PFOS degradation and highlights the importance of hydroxyl radicals in subsequent transformation steps. Consequently, the observed decrease in degradation efficiency upon adding TBA confirms the pivotal role of •OH in the overall piezocatalytic degradation process, while the effects of EDTA and LAA suggest auxiliary roles for H^+^ and •O2^−^ in generating •OH and facilitating PFOS breakdown.

As shown in Figure 4B–D, in the presence of TBA and EDTA, there is a minimal generation of PFOA and PFCAs, confirming that PFOS is not being decomposed in the absence of •OH and H^+^. Intriguingly, when LAA is introduced, the production of shorter chain PFCAs surpasses that observed without scavengers. Unlike the scenario without scavengers, where the concentration of degradation products begins to decrease during treatment, the presence of LAA leads to a saturation point after 2 h.

### 2.5 Practical Implications

**Figure 5:**
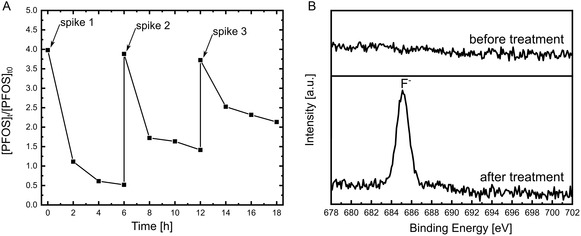
A) Time‐dependent decomposition profile with PFOS spikes to evaluate the reusability of BTO nanoparticles. B) F 1s XPS spectra of BTO nanoparticles before and after treatment.

To assess the performance over extended use of the BTO nanoparticles, three piezocatalytic degradation cycles of PFOS were conducted, where ultrasound activation was extended to 12 and 18 h. After each cycle, the nanoparticles were collected via centrifugation and dispersed in a fresh 4 mg L^−1^ PFOS solution. As depicted in **Figure** **5**A, the final concentrations achieved after each cycle were recorded as 0.5, 1.4, and 2.1 mg L^−1^. Notably, the rate constant for PFOS exhibited a significant decrease in the second and third cycles, resulting in an overall reduction in degradation of ≈54%. The addition of nanoparticles into a fresh PFOS solution in each cycle eliminated the possibility of smaller chain products accumulating and causing a decrease in performance. Consequently, further investigation was pursued by collecting the BTO nanoparticles to examine the potential saturation of their surface with adsorbed PFOS or PFCAs. For this purpose, XPS measurements were conducted (Figure S12, Supporting Information). The only visible difference in spectra between the nanoparticles before and after treatment was the presence of a peak at F 1s. As shown in Figure 5B, no peak is visible before treatment; however, after treatment, a distinct peak centering at 685 eV emerges. Given that no peaks were identified visible around 688 eV, which commonly indicates the presence of the C—F bond, this peak was ascribed to the negatively charged fluoride ions.^[^ ^43^ ^]^ In this regard, the adsorbed F^−^ also contributes to the incomplete mass balance discussed above. In addition, it is speculated that surface passivation competitively inhibits adsorption of the target PFAS, thereby reducing interactions with the generated ROS.

In the realm of water treatment, it is crucial to assess real‐world conditions where water often contains various inorganic ions and organic matter constituents. To investigate the practical applicability of piezocatalysis in degrading PFOS, experiments were conducted using lake water from Rotsee, Switzerland, and wastewater treatment plant effluent (WWTP) from Palapye, Botswana, both spiked with PFOS at a concentration of 4 mg L^−1^ using 1 g L^−1^ of BTO nanoparticles. The characteristics of lake water and sewage effluent are outlined in Table S2 (Supporting Information). In comparison with deionized (DI) water, both lake water and sewage effluent showed reduced removal efficiencies, likely due to the presence of natural organic matter and suspended solids acting as scavengers for ROS, thus impeding the degradation of target contaminants.^[^ ^44^ ^]^ Despite these challenges, as depicted in Figure S13 (Supporting Information), PFOS degradation in lake water and WWTP effluent reached ≈30% and 50%, respectively, highlighting its potential application in natural water matrices. Nevertheless, transitioning from laboratory‐scale studies with controlled conditions to real‐world scenarios necessitates careful consideration of the water matrix composition and its impact on treatment processes. Additional investigations should be conducted to examine the specific impact of inorganic cations and anions found in natural waters.

## 3 Conclusion

Our study addresses the urgent need for effective water treatment strategies to combat the environmental and health hazards posed by PFAS. Through piezocatalysis using BTO nanoparticles under ultrasound irradiation, we successfully achieved substantial degradation of PFOS in water, attaining a remarkable 90.5% removal after 6 h of treatment. Furthermore, the quantification of degradation products provided valuable insights into the transformation pathways of PFOS, shedding light on the formation and further degradation of shorter chain PFCAs. Continued research in this field is crucial to further validate the efficacy of piezocatalytic technologies for real‐world applications, paving the way for sustainable solutions to PFAS contamination challenges. Further investigation of alternative piezoelectric materials, novel mechanical activation sources, and synergistic combinations with sonocatalysis or piezocatalysis holds promise for optimizing the efficiency of the piezocatalytic degradation process aimed at PFAS removal.

## 4 Experimental Section

Chemicals, materials, and data analysis are presented in the Supporting Information.

4.1

4.1.1

##### BaTiO_3_ Nanoparticle Synthesis

To manufacture BaTiO_3_ nanoparticles, we first produced precursor H_2_Ti_3_O_7_ nanowires by mixing 0.7987 g of TiO_2_ with 35 mL of 10 M NaOH solution. The mixture was then transferred into an autoclave and heated for 24 h at 240 °C. The resulting nanowires were washed 3 times with water and 0.2 M hydrochloric acid solution (HCl) and soaked for 6–12 h in HCl with the same concentration. The nanowires were then washed again with DI water until reaching a pH of 7, followed by drying at 80 °C. In the second hydrothermal step, barium was introduced to form the nanoparticles. 35 mL of degassed DI water was prepared by bubbling with N_2_ for 10 min. Then, 2.208 g of Ba(OH)_2_·8H_2_O was added, and the mixture was stirred and sonicated until complete dispersion. Next, 0.2576 g of H_2_Ti_3_O_7_ precursor was added and dispersed in the mixture. The resulting suspension was transferred into an autoclave and heated to 240 °C for 12 h. The synthesized BTO nanoparticles were washed with a 0.2 M HCl solution and DI water, followed by drying at 80 °C.

##### Material Characterization

SEM was performed using a Zeiss ULTRA 55 instrument equipped with an EDX detector to determine the elemental composition of the samples. The samples were drop‐casted onto a silicon wafer and coated with a thin layer of palladium and gold to ensure conductivity before SEM analysis. Images were taken at 5 eV, with a 30 aperture and 10 mm working distance.

Raman inVia Renishaw confocal microscope was employed using a 514 nm excitation wavelength. The spectral acquisition conditions were: objective 50× (NA = 0.75), wavelength 514 nm, laser power 4.8 mW, exposure time 2 s, and 5 accumulations using a grating with 1800 grooves per millimeter.

XRD measurements were performed on an Empyrean diffractometer with a copper X‐ray source (*λ* = 1.5406 Å) and a PIXcel detector (Malvern Panalytical). The dispersion of nanoparticles in ethanol was drop‐casted onto a silicon wafer and let to air dry.

XPS experiments were performed in ESFOSCAN at CCiTUB, an equipment based on the PHI 5000 VersaProbe 4 instrument from Physical Electronics (ULVAC‐PHI). The measurements have been done with a monochromatic focused X‐ray source (aluminum Kα line of 1486.6 eV) calibrated using the 3d_5/2_ line of Ag with a full width at half maximum of 0.6 eV. The analyzed area was a circle of 100 μm of diameter, samples placed at 45° with respect to the analyzer axis, and the selected resolution for the spectra was 224 eV of pass energy and 0.8 eV/step for the general spectra, and 27 eV of pass energy and 0.1 eV/step for the high‐resolution spectra of the selected elements. A combination of low energy electron and ion (Ar^+^) guns (both less than 5 eV) was used to discharge samples if necessary. Measurements are referenced to the C 1s signal, whose binding energy is equal to 284.8 eV in adventitious carbon (from atmospheric contamination). Measurements were made in an ultrahigh vacuum chamber at a pressure between 5 E^−10^ and 5 E^−9 ^Torr.

##### Piezocatalysis Degradation Experiments

A 50 mL PFOS solution (4 mg L^−1^) was used for all degradation experiments with a 1 mg mL^−1^ BTO nanoparticle concentration. The ultrasonic treatment was administered using the Sonocool 255 (Bandelin) ultrasonic bath, operating at a frequency of 35 kHz, while maintaining a constant temperature of 24 °C and applying a power of 135 W (75% of total power). Before each experiment the ultrasound was degassed for 10 min. The 100 mL glass beaker was always placed in the center position, covered, and held in place with a rack (see Figure S1, Supporting Information). To achieve the desired 4 mg L^−1^ concentration, the PFOS solution was prepared by diluting a stock solution of 3200 ppm. This dilution was accomplished by adding 1.25 mL of the PFOS stock solution to 1 L of deionized water. The resulting solution was stirred overnight prior to separation into 50 mL glass vials and subsequently frozen. Before each experiment, the vials were thawed at room temperature. Subsequently, the nanoparticles were added and stirred for an additional 30 min in darkness to attain absorption–desorption equilibrium. All degradation experiments were conducted over a total time span of 6 h, during which 1 mL aliquots were collected every 2 h. To ensure the separation of the nanoparticles, the collected aliquots underwent two rounds of centrifugation at 14 000 rpm, separated, before being stored in the freezer for subsequent analysis. Control experiments were conducted to confirm that no degradation occurred in the absence of nanoparticles or ultrasound. Prior to initiating the experiments, PFOS adsorption on the nanoparticles was quantified by stirring the dispersion in dark for 60 min and time aliquots were taken before and after for analysis. In addition, a 4 mg L^−1^ PFOS solution without nanoparticles was also exposed to 6 h of ultrasound vibrations.

For the scavenger experiments, after thawing the prepared PFOS solution, 0.1 mM of TBA (pH = 5.66), EDTA (pH = 4.80), and LAA (pH = 3.45) were mixed in before the addition of the BTO nanoparticles.

For the cycling experiments, at the end of the 6 h treatment the BTO dispersion in PFOS solution was centrifuged at 11 000 rpm for 30 min and the isolated pellet was left to dry in the oven at 60 °C overnight. The catalyst was weighed and reused with a new 4 mg L^−1^ PFOS solution.

To investigate the decomposition of PFOS, the concentration of the initial product and its degradation products as well as the fluoride ions were determined using a Waters Acquity ultraperformance liquid chromatography system coupled with tandem mass spectrometry and an ion chromatography system (881 Compact IC pro), respectively. All analytical methods and data analysis are described in Text S2 (Supporting Information).

## Conflict of Interest

The authors declare no conflict of interest.

## Supporting information

Supplementary Material

## Data Availability

The data that support the findings of this study are available from the corresponding author upon reasonable request.

## References

[1] D. Ackerman Grunfeld , D. Gilbert , J. Hou , A. M. Jones , M. J. Lee , T. C. G. Kibbey , D. M. O’Carroll , Nat. Geosci. 2024, 17, 340.

[2] T. H. Begley , K. White , P. Honigfort , M. L. Twaroski , R. Neches , R. A. Walker , Food Addit. Contam. A 2005, 22, 1023. 10.1080/02652030500183474 16227186

[3] C. Baduel , C. J. Paxman , J. F. Mueller , J. Hazard. Mater. 2015, 296, 46. 25966923 10.1016/j.jhazmat.2015.03.007

[4] P. L. Lallas , Am. J. Int. Law 2001, 95, 692.

[5] P. Zareitalabad , J. Siemens , M. Hamer , W. Amelung , Chemosphere 2013, 91, 725. 23498059 10.1016/j.chemosphere.2013.02.024

[6] K. Kannan , S. Corsolini , J. Falandysz , G. Fillmann , K. S. Kumar , B. G. Loganathan , M. A. Mohd , J. Olivero , N. Van Wouwe , J. H. Yang , K. M. Aldous , Environ. Sci. Technol. 2004, 38, 4489. 15461154 10.1021/es0493446

[7] R. Monroy , K. Morrison , K. Teo , S. Atkinson , C. Kubwabo , B. Stewart , W. G. Foster , Environ. Res. 2008, 108, 56. 18649879 10.1016/j.envres.2008.06.001

[8] M. Reinhardt , R. Kozel , S. Zimmermann , H. Rupp , O. Zoller , E. Hoehn , Iahs‐Aish P 2011, 342, 112.

[9] V. Barry , A. Winquist , K. Steenland , Environ. Health Persp. 2013, 121, 1313. 10.1289/ehp.1306615 PMC3855514 24007715

[10] A. M. Ingelido , V. Marra , A. Abballe , S. Valentini , N. Iacovella , P. Barbieri , M. G. Porpora , A. Di Domenico , E. De Felip , Chemosphere 2010, 80, 1125. 20633921 10.1016/j.chemosphere.2010.06.025

[11] N. M. Crawford , S. E. Fenton , M. Strynar , E. P. Hines , D. A. Pritchard , A. Z. Steiner , Reprod. Toxicol. 2017, 69, 53. 28111093 10.1016/j.reprotox.2017.01.006 PMC5690561

[12] A. L. Ling , Sci. Total Environ. 2024, 918, 170647. 38325453 10.1016/j.scitotenv.2024.170647

[13] H. Smaili , C. Ng , Environ. Sci.‐Wat. Res. 2023, 9, 344.

[14] S. Das , A. Ronen , Membranes 2022, 12, 662. 35877866 10.3390/membranes12070662 PMC9325267

[15] A. Mojiri , J. L. Zhou , N. Ozaki , B. KarimiDermani , E. Razmi , N. Kasmuri , Chemosphere 2023, 330, 138666. 37068615 10.1016/j.chemosphere.2023.138666

[16] Q. Tang , J. Wu , D. Kim , C. Franco , A. Terzopoulou , A. Veciana , J. Puigmartí‐Luis , X. Z. Chen , B. J. Nelson , S. Pané , Adv. Funct. Mater. 2022, 32, 2202180.

[17] J. Wu , Q. Xu , E. Z. Lin , B. W. Yuan , N. Qin , S. K. Thatikonda , D. H. Bao , ACS Appl. Mater. Interfaces 2018, 10, 17842. 29726250 10.1021/acsami.8b01991

[18] D. Masekela , N. C. Hintsho‐Mbita , S. Sam , T. L. Yusuf , N. Mabuba , Arab. J. Chem. 2023, 16, 104473.

[19] F. Mushtaq , X. Z. Chen , M. Hoop , H. Torlakcik , E. Pellicer , J. Sort , C. Gattinoni , B. J. Nelson , S. Pané , Iscience 2018, 4, 236. 30240743 10.1016/j.isci.2018.06.003 PMC6146592

[20] X. L. Xu , Y. M. Jia , L. B. Xiao , Z. Wu , Chemosphere 2018, 193, 1143. 29874742 10.1016/j.chemosphere.2017.11.116

[21] X. E. Ning , A. Z. Hao , Y. L. Cao , J. D. Hu , J. Xie , D. Z. Jia , J. Colloid Interface Sci. 2020, 577, 290. 32485412 10.1016/j.jcis.2020.05.082

[22] Y. Bai , J. Z. Zhao , Z. L. Lv , K. Lu , J. Mater. Sci. 2020, 55, 14112.

[23] Q. Tang , J. Wu , X. Z. Chen , R. Sanchis‐Gual , A. Veciana , C. Franco , D. Kim , I. Surin , J. Pérez‐Ramfrez , M. Mattera , A. Terzopoulou , N. Qin , M. Vukomanovic , B. J. Nelson , J. Puigmartf‐Luis , S. Pan , Nano Energy 2023, 108, 108202.

[24] R. N. Guo , L. Li , Z. W. Zhao , S. Zhang , J. Hazard. Mater. 2024, 465, 133040. 38029588 10.1016/j.jhazmat.2023.133040

[25] N. Y. Yang , S. S. Yang , Q. Q. Ma , C. Beltran , Y. Q. Guan , M. Morsey , E. Brown , S. Fernando , T. M. Holsen , W. Zhang , Y. Yang , Environ. Sci. Technol. 2023, 10, 198. 10.1021/acs.estlett.2c00902 PMC10074478 37034438

[26] Z. Deng , Y. Dai , W. Chen , X. M. Pei , J. H. Liao , Nanoscale Res. Lett. 2010, 5, 1217. 20596350 10.1007/s11671-010-9629-7 PMC2894195

[27] R. X. Wang , Q. Zhu , W. S. Wang , C. M. Fan , A. W. Xu , New J. Chem. 2015, 39, 4407.

[28] Y. P. Ma , H. Luo , X. F. Zhou , R. Guo , F. Dang , K. C. Zhou , D. Zhang , Nanoscale 2020, 12, 8230. 32129360 10.1039/c9nr08572f

[29] G. G. Guan , G. J. Gao , J. Xiang , J. N. Yang , L. Gong , X. Chen , Y. M. Zhang , K. Y. Zhang , X. F. Meng , ACS Appl. Nano Mater. 2020, 3, 8424.

[30] S. J. Smith , M. Lauria , C. P. Higgins , K. D. Pennell , J. Blotevogel , H. P. H. Arp , Environ. Sci. Technol. 2024, 58, 2587. 38314573 10.1021/acs.est.3c10617 PMC10867837

[31] J. Horst , J. McDonough , I. Ross , E. Houtz , Ground Water Monit. R 2020, 40, 17.

[32] G. Prasanna , H. D. P. Nguyen , S. Dunn , A. Karunakaran , F. Marken , C. R. Bowen , B. N. T. Le , H. D. Nguyen , T. P. T. Pham , Nano Energy 2023, 116, 108794.

[33] F. Bössl , I. Tudela , Curr. Opin. Green Sust. 2021, 32, 100537.

[34] M. G. Evich , M. J. B. Davis , J. P. McCord , B. Acrey , J. A. Awkerman , D. R. U. Knappe , A. B. Lindstrom , T. F. Speth , C. Tebes‐Stevens , M. J. Strynar , Z. Wang , E. J. Weber , W. M. Henderson , J. W. Washington , Science 2022, 375, 10.1126/science.abg9065. PMC8902460 35113710

[35] T. Teymourian , T. Teymoorian , E. Kowsari , S. Ramakrishna , Res. Chem. Intermed. 2021, 47, 4879.

[36] Y. Deng , Z. H. Liang , X. W. Lu , D. Chen , Z. Li , F. Wang , Chemosphere 2021, 283, 10.1016/j.chemosphere.2021.131168. 34182635

[37] M. B. Starr , X. D. Wang , Sci. Rep. 2013, 3, 10.1038/srep02160. PMC3703609 23831736

[38] L. Duan , B. Wang , K. Heck , S. Guo , C. A. Clark , J. Arredondo , M. Wang , T. P. Senftle , P. Westerhoff , X. Wen , Y. Song , M. S. Wong , Environ. Sci. Technol. Lett. 2020, 7, 613.

[39] Z. Song , X. L. Dong , N. Wang , L. H. Zhu , Z. H. Luo , J. D. Fang , C. H. Xiong , Chem. Eng. J. 2017, 317, 925.

[40] R. K. Singh , S. Fernando , S. F. Baygi , N. Multari , S. M. Thagard , T. Holsen , Abstr. Pap. Am. Chem. S 2019, 258. 10.1021/acs.est.8b07031 30768259

[41] J. Radjenovic , N. Duinslaeger , S. S. Avval , B. P. Chaplin , Environ. Sci. Technol. 2022, 56, 18079. 36458333 10.1021/acs.est.2c08354

[42] J. F. Niu , Y. Li , E. X. Shang , Z. S. Xu , J. Z. Liu , Chemosphere 2016, 146, 526. 26745381 10.1016/j.chemosphere.2015.11.115

[43] K. L. Zhang , J. Huang , G. Yu , Q. W. Zhang , S. B. Deng , B. Wang , Environ. Sci. Technol. 2013, 47, 6471. 23676146 10.1021/es400346n

[44] J. L. Wang , S. Z. Wang , Chem. Eng. J. 2021, 411, 128392.

