# Supplementary material for: Breaking the Perfluorooctane Sulfonate Chain: Piezocatalytic Decomposition of PFOS Using BaTiO3 Nanoparticles
Source: Small Sci. 2024 Aug 28;4(12):2400337. doi: 10.1002/smsc.202400337 (PMC11935004; doi:10.1002/smsc.202400337)
Supplement: Supplementary file 1 — Supplementary Material [file SMSC-4-2400337-s001.pdf]

Supporting Information

Breaking the Perfluorooctane Sulfonate Chain: Piezocatalytic Decomposition of PFOS Using $BaTiO_3$ Nanoparticles

Andrea Veciana, Sarah Steiner, Qiao Tang, Vitaly Pustovalov, Joaquin Llacer-Wintle, Jiang Wu, Xiang-Zhong Chen, Trust Manyiwa, Venecio U. Ultra Jr., Beltzane Garcia-Cirera, Josep Puigmartí-Luis, Carlos Franco, David J. Janssen, Laura Nyström, Samy Boulos*, Salvador Pané*

A. Veciana, S. Steiner, Q. Tang, V. Pustovalov, J. Wu, C. Franco, S. Pané Institute of Robotics and Intelligent Systems ETH Zurich, Tannenstrasse 3, Zurich CH 8092, Switzerland

David Janssen
Eawag: Swiss Federal Institute of Aquatic Science & Technology, Department Surface Waters, 6047 Kastanienbaum, Switzerland

Trust Manyiwa, Venecio U. Ultra Jr.,
Earth and Environmental Sciences, Faculty of Science, Botswana International University of Science and Technology, Palapye, Botswana

L. Nyström, S. Boulos
Laboratory of Food Biochemistry, Department of Health Sciences and Technology, ETH Zurich, Schmelzbergstrasse 9, 8092 Zürich, Switzerland.

X.-Z. Chen
Institute of Optoelectronics, State Key Laboratory of Photovoltaic Science and Technology, Shanghai Frontiers Science Research Base of Intelligent Optoelectronics and Perception, International Institute of Intelligent Nanorobots and Nanosystems, Fudan University, Shanghai 200433, People's Republic of China
Yiwu Research Institute of Fudan University
Yiwu, Zhejiang 322000, People's Republic of China

Josep Puigmartí-Luis[1,2], Beltzane Garcia-Cirera[1]
[1]Departament de Ciència de Materials i Química Física, Institut de Química Teòrica i Computacional, Universitat de Barcelona, Barcelona 08028, Spain
[2]Institució Catalana de Recerca i Estudis Avançats (ICREA)
Pg. Lluís Companys 23, Barcelona 08010, Spain

Text S1. Materials and Methods

All materials were used as purchased. Barium hydroxide octahydrate ($Ba(OH)_2 \cdot H_2O$, CAS-No. 12230-71-6, Product No. 21573), sodium hydroxide (NaOH, 98%, CAS No. 1310-73-2, Product No. S5881), titanium(IV) oxide (TiO_2, 99.5%, CAS No., Product No. 718467), perfluorooctanesulfonic acid (PFOS, CAS No. 1763-23-1, Product No. 77283) were purchased from Sigma-Aldrich. Formic acid, acetonitrile (ACN), 2-propanol, and water (all LC-MS grade) were purchased from Fisher Scientific AG (Reinach, Switzerland). Ammonium acetate (LC-MS grade) was from Sigma-Aldrich Chemie GmbH (Germany). Analytical standards were purchased from Wellington Laboratories.

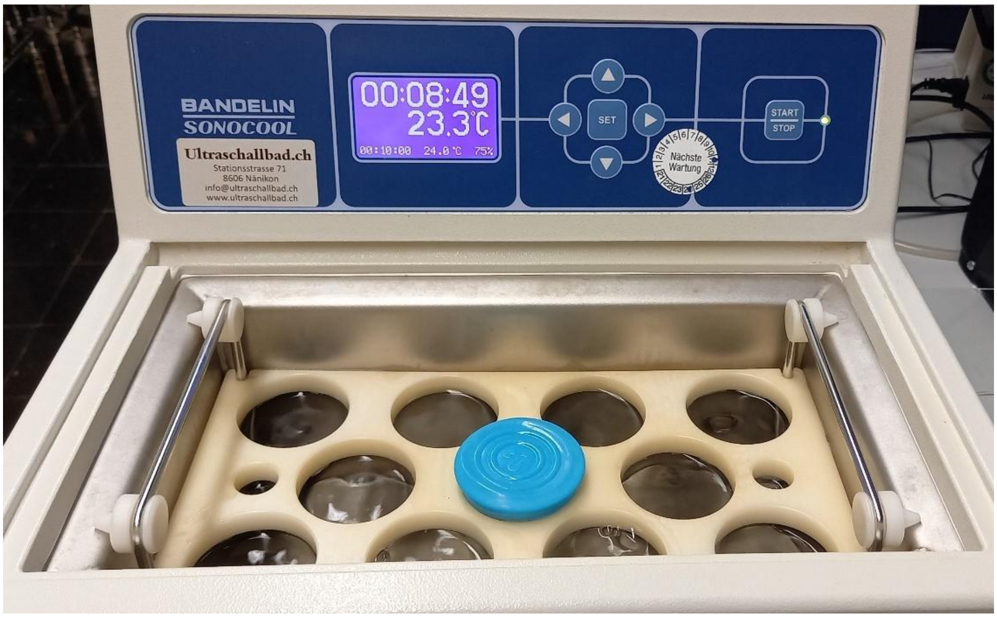

Figure S1. Piezocatalysis set-up (with lid removed) using the Sonocool 255 (Bandelin) ultrasonic bath, operating at a frequency of 35 kHz, while maintaining a constant temperature of 24 °C and applying a power of 135 W (75% of total power).

Text S2. Analytical Methods

2.1 UPLC/MS

Ultraperformance liquid chromatography coupled to a mass spectrometer (UPLC-MS) was used to follow the degradation of PFOS during treatments. Each sample underwent a 50% dilution using a 60% methanol:40% water solution, achieved by adding 0.75 mL of the sample and 0.75 mL of MeOH/H_2O. Subsequently, the diluted samples were transferred to 1.5 mL short-thread vials and sealed with septa silicone/PTFE screw caps.

A Waters Acquity UPLC system with Acquity UPLC BEH C18 column (50 mm × 2.1 mm, 1.7 μm particle size) was used, coupled to a Synapt G2 quadrupole time-of-flight (qToF) analyzer with electrospray ionization (ESI) (Waters Corp., Milford, MA, USA).

Chromatographic conditions: Eluent A was composed of 2 mM ammonium acetate in water (pH = 6), and eluent B was pure acetonitrile (ACN). The flow rate was 0.2 mL/min at 40 °C, with a total run time of 14 min and an injection volume of 5 μL in partial loop mode. Gradient started isocratically with 70% eluent A/ 30% B for 1 min, followed by a linear gradient to 50% A within 6 min, going further down to 2% A within 2 min, staying there for 1 min, to linearly go up again 70% A within 0.5 min, and staying at the initial conditions for 3.5 min. Strong wash solvent was pure 2-propanol, and the weak wash and seal wash were 30% ACN and 10% ACN in water, respectively (all MS-grade solvents).

MS conditions: Before each run, the MS was freshly calibrated with a 5 mM sodium formate solution (in 2-propanol/water 9:1) for the *m*/*z* 50–1200 range. A leucine-enkephalin solution (2 ng μL^{-1} in 1:1 ACN/water + 0.1% formic acid) was used as the lockmass (*m*/*z* 554.2615), which was acquired every 60 s during the UPLC-MS measurements (scan time of 0.3 s; correction applied with 5 scans averaged; lockspray capillary voltage of 2.2 kV). The spectra were acquired in resolution and negative ionization mode. The capillary, sample cone, and extraction cone voltages were set at 1.5 kV, 35 V, and 4 V, respectively. The desolvation gas flow rate was 850 L h^{-1} at 450 °C. The cone gas flow was 20 L h^{-1} and the source temperature was 120 °C. Full scan mass spectra were acquired from *m*/*z* 50 to 1200 with scan times of 1.0 s in centroid mode. The perfluorinated carboxylic acids exhibited partial in-source fragmentation, with the respective decarboxylated fragment ion $[M - CO_2 - H]^-$ being more abundant than the pseudo-molecular ion $[M - H]^-$. Hence, for the carboxylates, the signal of this fragment ion was used for quantification to achieve higher sensitivity. For PFOS, there was no significant in-source fragmentation.

The calibration curve for PFOS was obtained by preparing a set of dilutions ranging from 0 to 2.5 mg L^{-1} using a prepared 4 mg/L stock solution. The measured data was fitted with polynomial fit y = Ax^2 + Bx + C, where C = 0, as seen in Figure S2. Using the coefficients

A and B, the corresponding concentrations were calculated through the quadratic equation. An individual calibration curve was measured for each set of measurements to account for intensity variations of the LC-MS.

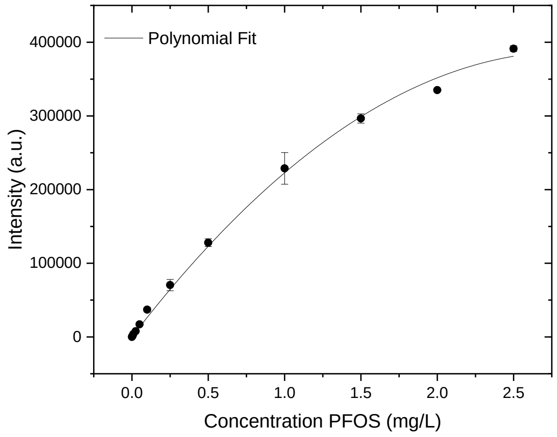

Figure S2. Calibration curve of PFOS solution.

Additionally, calibrations standards listed in Table S1 were used to prepare calibration curves for PFOA, PFHpA, PFHxA, PFPeA, and PFBA. The measured data was fitted with a linear fit y = mx + b, as seen in Figure S3.

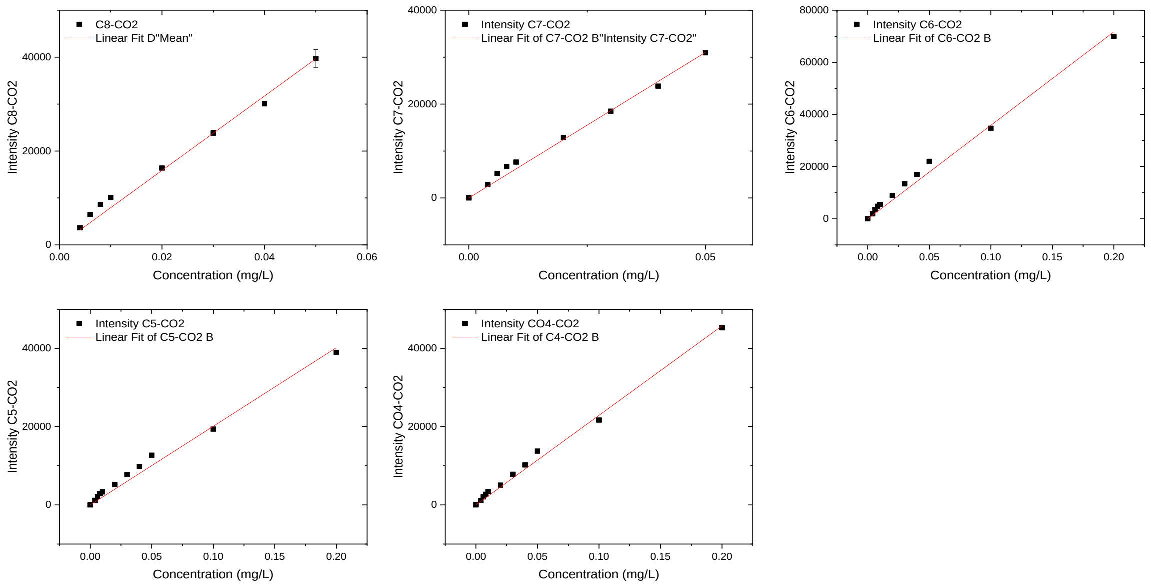

Figure S3. Calibration curve of PFC-MXA calibration standard.

Table S1. acronym, full name, CAS numbers, supplier, purity, and concentration of analytical standards.

acronym	analytical standard	CAS number	supplier, purity, and concentration
PFBA	perfluoro-*n*-butanoic acid	375-22-4 (acid)	
PFPeA	perfluoro-*n*-pentanoic acid	2706-90-3 (acid)	
PFHxA	perfluoro-*n*-hexanoic acid	307-24-4 (acid)	PFC-MXA (mixture)
PFHpA	perfluoro-*n*-heptanoic acid	375-85-9 (acid)	Wellington
PFOA	perfluoro-*n*-octanoic acid	335-67-1 (acid)	Laboratories,
PFNA	perfluoro-*n*-nonanoic acid	375-95-1 (acid)	> 98 %
PFDA	perfluoro-*n*-decanoic acid	335-76-2 (acid)	2.0 µg/mL ± 5 % in
PFUDA	perfluoro-*n*-undecanoic acid	2058-94-8 (acid)	methanol of each
PFDoA	perfluoro-*n*-dodecanoic acid	307-55-1 (acid)	compound
PFTrDA	perfluoro-*n*-tridecanoic acid	72629-94-8 (acid)	
PFTeDA	perfluoro-n-tetradecanoic acid	376-06-7 (acid)	

2.2 IC

The concentration of fluoride ions was determined using an ion chromatography system (881 Compact IC pro) in conjunction with the 942 Eluent Production Module and the 889 Sample Center, all supplied by Metrohm Schweiz AG. For the chromatographic separation, a Metrosept A Supp 5 100/4.0 column from Metrohm Schweiz AG was employed. The analysis was conducted at a flow rate of 0.9 mL min-1 and using an eluent consisting of a mixture of 1 mM sodium bicarbonate and 3.2 mM sodium carbonate dissolved in 1 liter of water. Calibration of the system was performed across a range of fluoride ion concentrations spanning from 25 µM to 250 µM F^-. To ensure the accuracy and reliability of the measurements, quality checks were conducted using a multi-element standard anion solution sourced from Carl-Roth AG (Part No. 2668.1).
The defluorination ratio is defined as:

$$deF\% = \frac{[F^-]}{[PFAS]_0 \times N_{C-F}} \times 100\% \qquad \text{(eq. S1)}$$

where $[F^-]$ is the measured concentration of F^- ions released in the solution, $[PFAS]_0$ is the initial concentration (in molar units) of PFOS and $N_{C\text{-}F}$ is the number of C-F bonds of PFOS.

Text S3. Supplementary Results

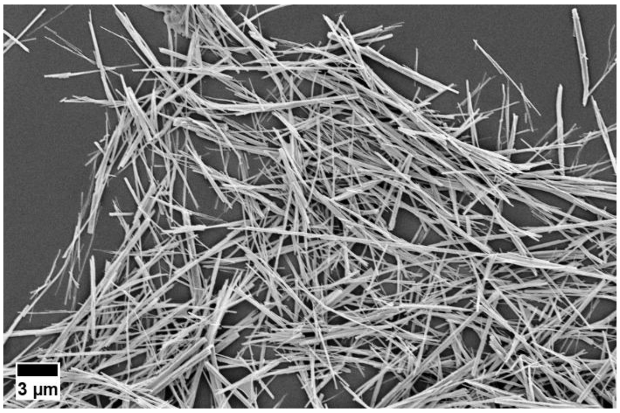

Figure S4. SEM images of $H_2Ti_3O_7$ nanowires used as precursor.

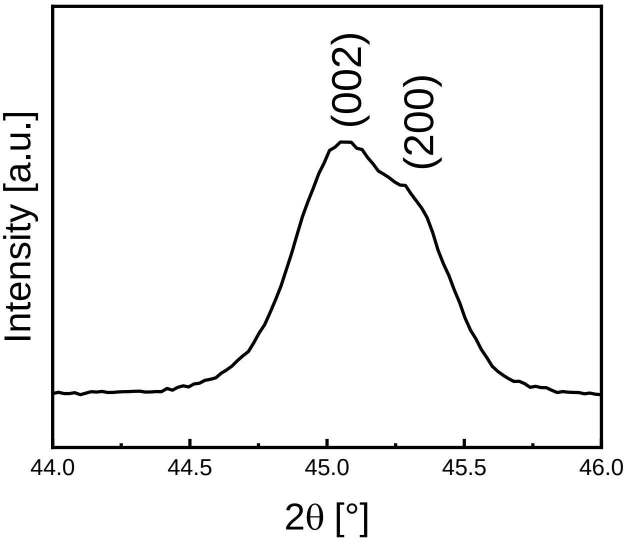

Figure S5. Magnification of the XRD peak of the BTO nanoparticles at $2\theta = 45°$ showcasing a peak split.

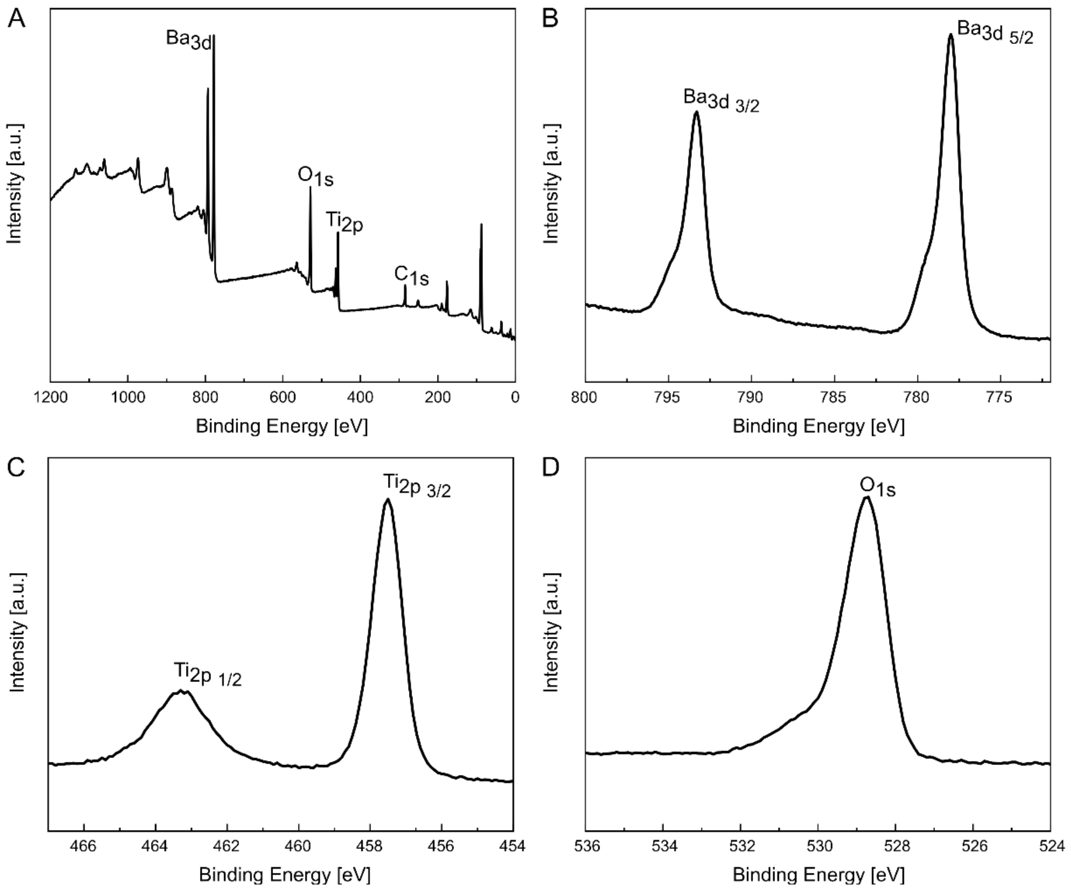

Figure S6. XPS spectra of BTO nanoparticles: (a) survey, (b) Ba 3d, (c) Ti 2p, and (d) O 1s.

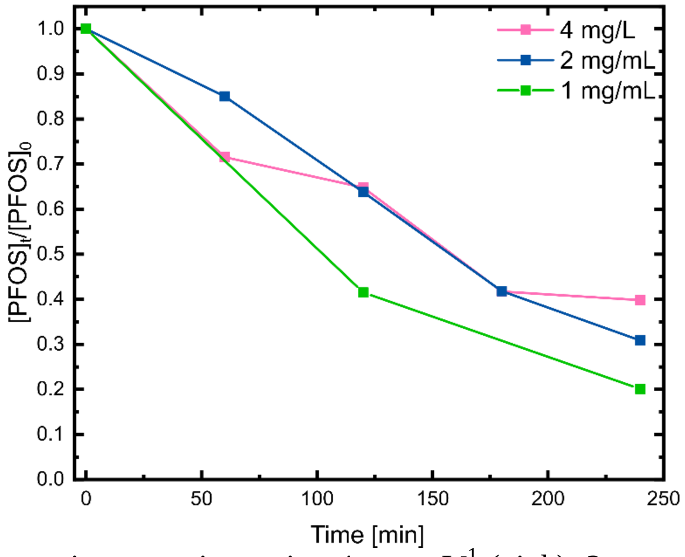

Figure S7. PFOS concentration over time using 4 mg mL^{-1} (pink), 2 mg mL^{-1} (blue), and 1 mg mL^{-1} (green) catalyst load.

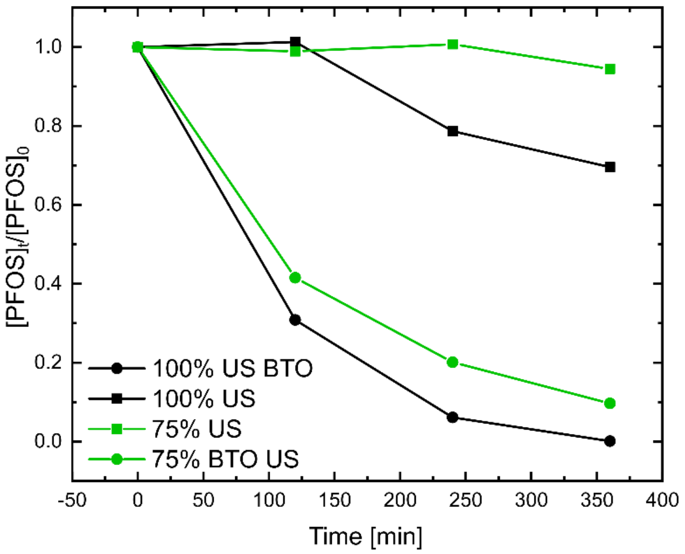

Figure S8. PFOS concentration over time using 100% (black) and 75% (green) ultrasound power (US, square), both with and without the presence of nanoparticles (BTO, circle).

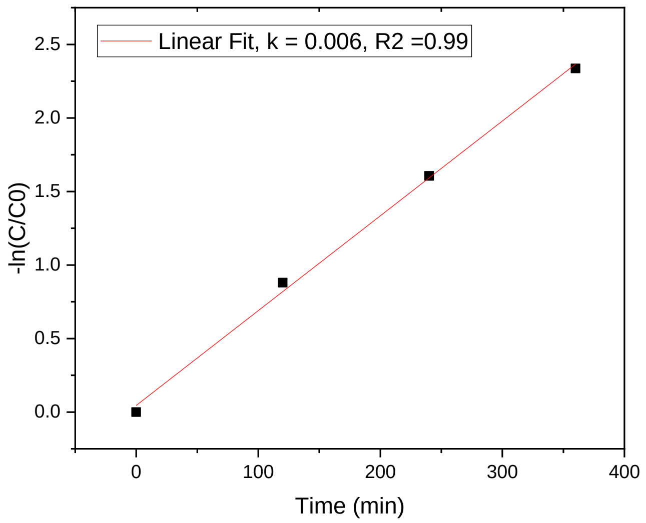

Figure S9. Pseudo-first order linear fit of the PFOS decay.

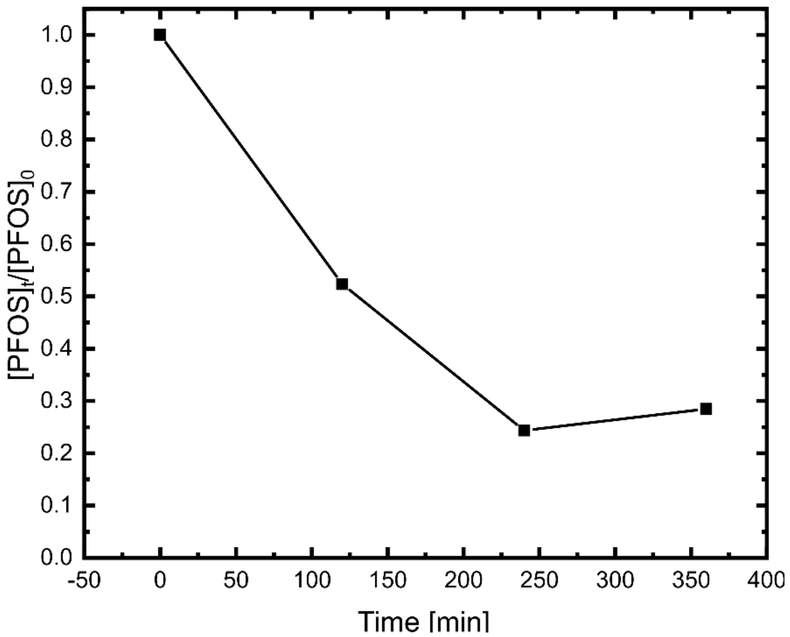

Figure S10. PFOS concentration over time, for the experiment with the 10x lower initial concentration of 0.4 mg L^{-1}.

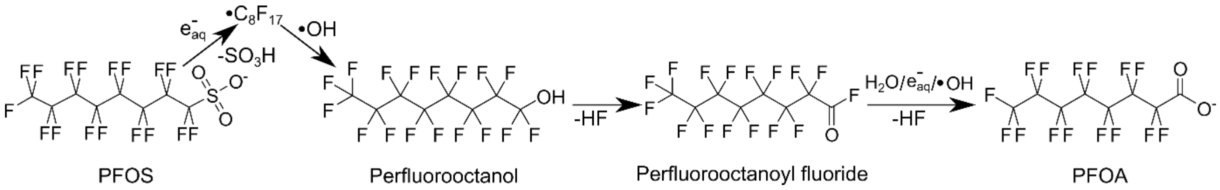

Figure S11. PFOS degradation pathway into PFOA as proposed by [1].

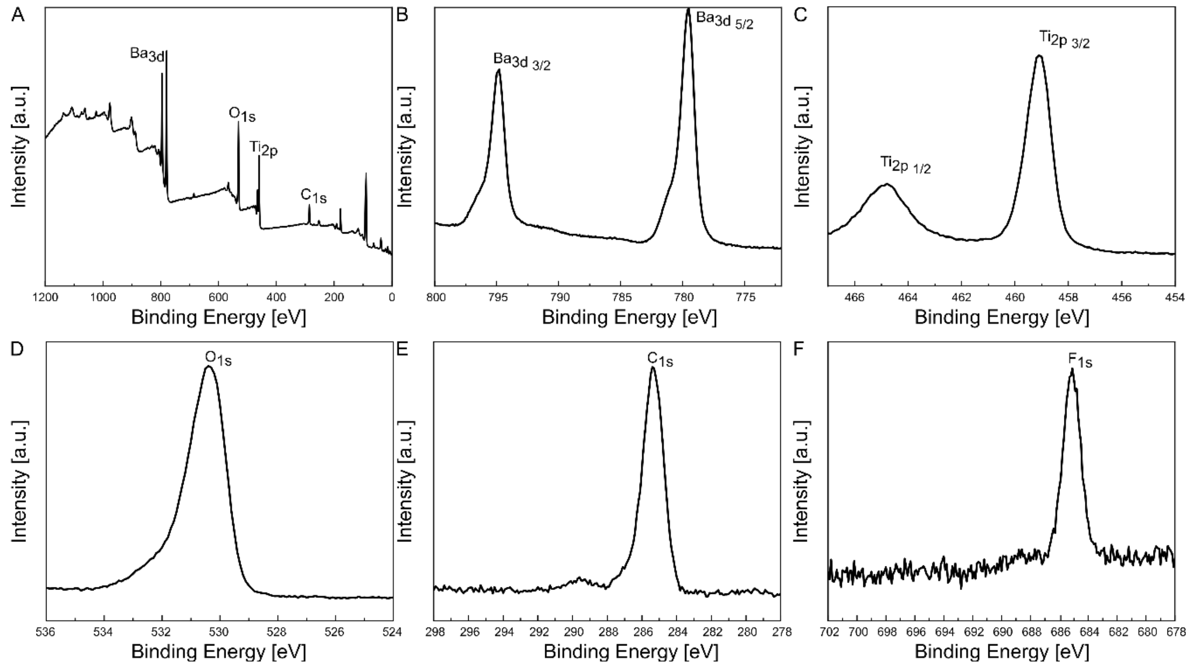

Figure S12. XPS spectra of BTO nanoparticles after treatment: (a) survey, (b) Ba 3d, (c) Ti 2p, (d) O 1s, (e) C 1s, and (f) F 1s.

Text S4. Testing Real Water

The real wastewater samples were collected from the effluent of BIUST's wastewater treatment plant in Botswana and the Rotsee in Switzerland and subsequently filtered through a GFF filter and frozen until use. The water samples were separated into 50 mL glass vials and spiked with 62.5 µL of the PFOS stock solution to reach the desired concentration of 4 mg L^{-1}.

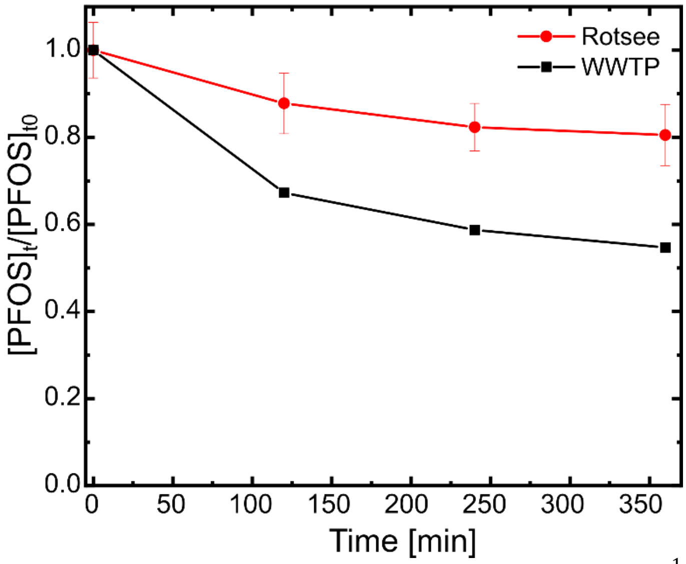

Figure S13. PFOS concentration over time, initial concentration 4 mg L^{-1}.

Table S2. Inorganic ions present in WWTP effluent and Rotsee samples before treatment.

Samples	IC Na^+	IC NH_4^+	IC K^+	IC Mn^{2+}	IC Ca^{2+}	IC Mg^{2+}	IC F^-	IC Cl^-	IC NO_2^-	IC Br^-	IC NO_3^-	IC PO_4^{3-}	IC SO_4^{2-}
	[µmol]	[µmol]	[µmol]	[µmol]	[µmol]	[µmol]	[µmol]	[µmol]		[µmol]	[µmol]	[µmol]	[µmol]
WWTP	2329	57	471	74	640	281	24	1338	25	LOQ <12.5	2285	98	256
Rotsee	128	<12.5	32.7	-	671	151	<12.5	95	0.11	< 12.5	2.65	0.04	138

References

1. Singh, R. K.; Fernando, S.; Baygi, S. F.; Multari, N.; Thagard, S. M.; Holsen, T., Breakdown products from perfluorinated alkyl substances (PFAS) degradation in a plasma-based water treatment process. *Abstr Pap Am Chem S* **2019,** *258,*
